# Supplementary material for: Association between Age of Onset of Hypertension and Incident Atrial Fibrillation
Source: J Pers Med. 2022 Jul 21;12(7):1186. doi: 10.3390/jpm12071186 (PMC9317856; doi:10.3390/jpm12071186)
Supplement: Supplementary file 1 [file jpm-12-01186-s001.zip › jpm-1815987-supplementary.pdf]

Supplementary Tables

Table S1. Multivariate models for newly developed AF

	Covariates	HR (95% CI)	p value
Model 1	HTN (vs. normotension)	1.93 (1.32-2.80)	0.001
	Age (year)	1.06 (1.04-1.08)	<0.001
	Female sex (vs. male sex)	0.59 (0.42-0.82)	0.002
	Urban residential area (vs. rural)	1.98 (1.34-2.94)	<0.001
	Low PA (vs. Intermediate PA)*	2.00 (1.29-3.08)	0.000
	High PA (vs. Intermediate PA)*	2.48 (1.46-4.22)	<0.001
Model 2	HTN onset age <45 years (vs. normotension)	3.18 (1.74-5.82)	<0.001
	HTN onset age 45-54 years (vs. normotension)	1.78 (0.98-3.23)	0.059
	HTN onset age 55-64 years (vs. normotension)	1.51 (0.81-2.82)	0.193
	HTN onset age ≥65 years (vs. normotension)	1.26 (0.39-4.08)	0.697
	Age (year)	1.07 (1.04-1.09)	<0.001
	Female sex (vs. male sex)	0.60 (0.43-0.83)	0.002
	Urban residential area (vs. rural)	1.98 (1.33-2.94)	<0.001
	Low PA (vs. Intermediate PA)*	1.99 (1.29-3.09)	0.002
	High PA (vs. Intermediate PA)*	2.47 (1.45-4.19)	<0.001

Covariates include age, sex, diabetes, dyslipidemia, body mass index, central obesity, smoking, alcohol, income ≥median, PA, myocardial infarction, congestive heart failure, thyroid disease, asthma, chronic lung diseases

Models were reduced through a backward variable selection procedure (cut-off criterion p>0.05)

* Where low PA is <20.0 MET-h/day, intermediate PA is 20.0-39.9 MET-h/day and high PA is ≥40 MET-h/day.

PA, physical activity; HTN, hypertension; MET, metabolic equivalent task; HR, hazard ratio; CI, confidence interval

Supplementary Figures

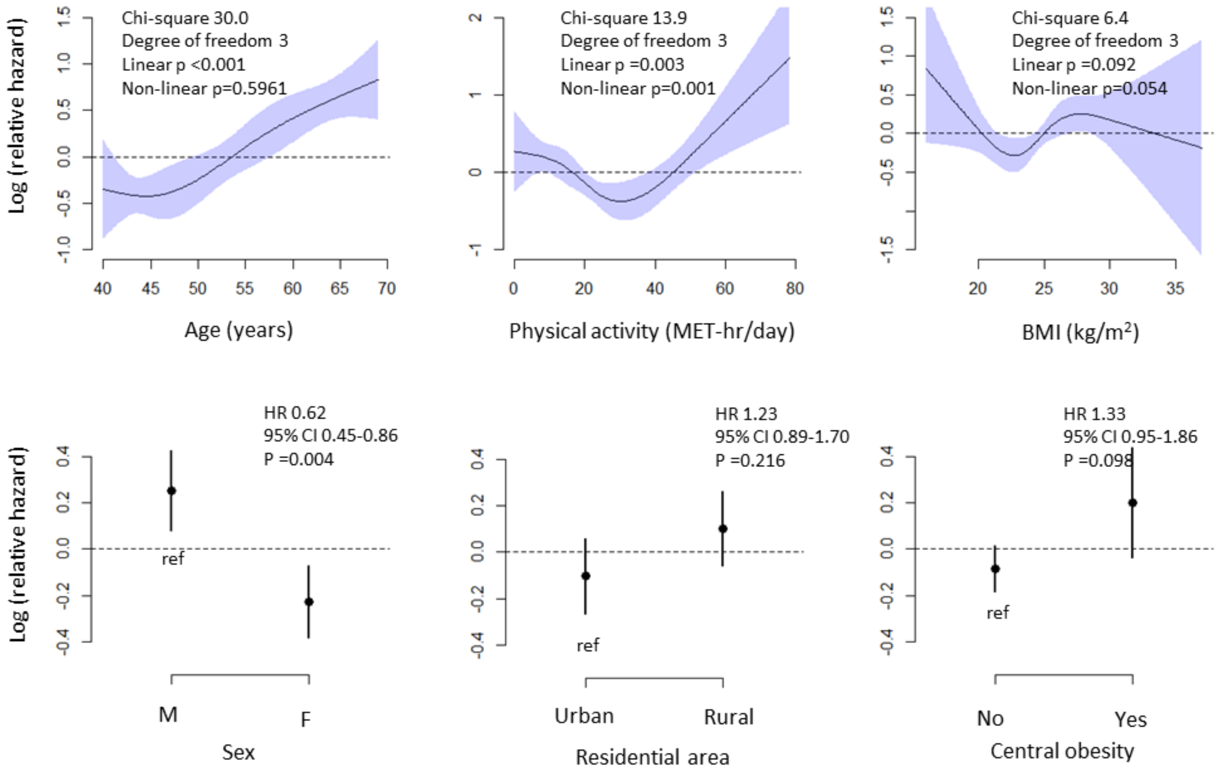

Figure S1. The risk of incident AF according to the covariates of the age of HTN onset.

Univariate Cox proportional hazards models were used to estimate the risk of incident AF according to covariates. The nonlinear models using a restrictive cubic spline fit showed that age was linearly associated with the risk of incident AF, whereas physical activity had a V-shaped biphasic relationship with the risk of incident AF. BMI and central obesity were marginally associated with the risk of incident AF but in opposite directions. Male sex was associated with an increased risk of incident AF, whereas the residential area was not associated with the risk of AF.

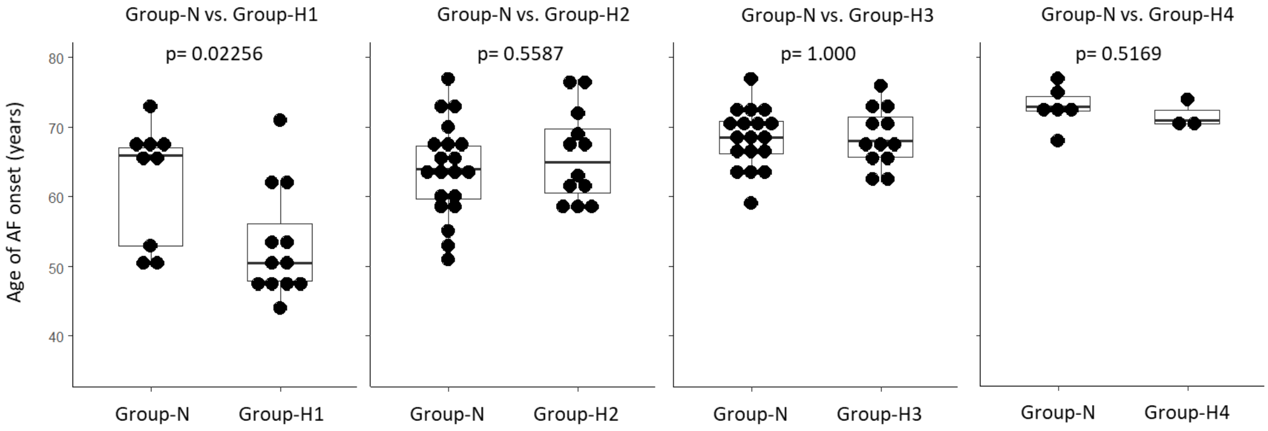

Figure S2. The propensity score matched-age of AF onset in the different groups.

The age of AF onset was significantly lower in Group-H1 compared with Group-N (normotensive age and sex-matched participants), but there was no significant difference between other hypertensive participant and Group-N.
